# Hyper-CVAD-Based Stem Cell Microtransplant as Post-Remission Therapy in Acute Lymphoblastic Leukemia

**DOI:** 10.1093/stcltm/szac066

**Published:** 2022-10-01

**Authors:** Bo Cai, Yi Wang, Yangyang Lei, Yanping Shi, Qiyun Sun, Jianhui Qiao, Kaixun Hu, Yaqing Lei, Bingxia Li, Tieqiang Liu, Zhiqing Liu, Bo Yao, Xuecong Zhao, Xiaofei Li, Wen Zhao, Xiujie Feng, Anli Xie, Xin Ning, Mingxing Feng, Weiwei Zhao, Jiayue Guo, Huisheng Ai, Changlin Yu, Mei Guo

**Affiliations:** Department of Hematology, The Fifth Medical Center, Chinese PLA General Hospital, Beijing, People’s Republic of China; Department of Hematology, The Fifth Medical Center, Chinese PLA General Hospital, Beijing, People’s Republic of China; Department of Hematology, The Fifth Medical Center, Chinese PLA General Hospital, Beijing, People’s Republic of China; Department of Hematology, The Fifth Medical Center, Chinese PLA General Hospital, Beijing, People’s Republic of China; Department of Hematology, The Fifth Medical Center, Chinese PLA General Hospital, Beijing, People’s Republic of China; Department of Hematology, The Fifth Medical Center, Chinese PLA General Hospital, Beijing, People’s Republic of China; Department of Hematology, The Fifth Medical Center, Chinese PLA General Hospital, Beijing, People’s Republic of China; Department of Hematology, The Fifth Medical Center, Chinese PLA General Hospital, Beijing, People’s Republic of China; Department of Hematology, The Fifth Medical Center, Chinese PLA General Hospital, Beijing, People’s Republic of China; Department of Hematology, The Fifth Medical Center, Chinese PLA General Hospital, Beijing, People’s Republic of China; Department of Hematology, The Fifth Medical Center, Chinese PLA General Hospital, Beijing, People’s Republic of China; Department of Hematology, The Fifth Medical Center, Chinese PLA General Hospital, Beijing, People’s Republic of China; Department of Hematology, The Fifth Medical Center, Chinese PLA General Hospital, Beijing, People’s Republic of China; Department of Hematology, The Fifth Medical Center, Chinese PLA General Hospital, Beijing, People’s Republic of China; Department of Hematology, The Fifth Medical Center, Chinese PLA General Hospital, Beijing, People’s Republic of China; Department of Hematology, The Fifth Medical Center, Chinese PLA General Hospital, Beijing, People’s Republic of China; Department of Hematology, The Fifth Medical Center, Chinese PLA General Hospital, Beijing, People’s Republic of China; Department of Hematology, The Fifth Medical Center, Chinese PLA General Hospital, Beijing, People’s Republic of China; Department of Hematology, The Fifth Medical Center, Chinese PLA General Hospital, Beijing, People’s Republic of China; Department of Hematology, The Fifth Medical Center, Chinese PLA General Hospital, Beijing, People’s Republic of China; Department of Clinical Medicine, Capital Medical University, Beijing, People’s Republic of China; Department of Hematology, The Fifth Medical Center, Chinese PLA General Hospital, Beijing, People’s Republic of China; Department of Hematology, The Fifth Medical Center, Chinese PLA General Hospital, Beijing, People’s Republic of China; Department of Hematology, The Fifth Medical Center, Chinese PLA General Hospital, Beijing, People’s Republic of China

**Keywords:** microtransplant, acute lymphoblastic leukemia, stem cells, cellular therapy, post-remission therapy, hyper-CVAD regimen, microchimerism, graft-versus-host disease

## Abstract

Post-remission strategies for patients with acute lymphoblastic leukemia (ALL) are limited to the multiagent chemotherapy and allogeneic stem cell transplant (allo-SCT), and cellular therapies are seldom involved. Although chemotherapy combined with mismatched granulocyte colony-stimulating factor mobilized peripheral blood mononuclear cell infusion (microtransplant, MST) has been studied in patients with acute myeloid leukemia, its efficacy in ALL is still undetermined. We enrolled 48 patients receiving hyper-CVAD-based MST between July 1, 2009, and January 31, 2018. No acute or chronic graft-versus-host disease occurred in patients receiving MST. Four-year overall survival (OS) and leukemia-free survival (LFS) were 62% and 35%, respectively, and the 4-year relapse rate was 65%. No patient experienced non–relapse mortality. Subgroup analysis showed that OS rates were comparable between groups with different age, risk stratification, minimal residual disease status prior to MST and immunophenotype. Adult patients tended to achieve better 4-year LFS (62% vs. 26%, *P* = .058) and lower hematologic relapse rate (38% vs. 74%, *P* = .058) compared with adolescent and young adult patients. Donor chimerism/microchimerism was detectable ranging from 0.002% to 42.78% in 78% (42/54) available samples within 14 days after each infusion and at 3 months or one year after the last cell infusion. Multivariate analyses demonstrated that white blood cells <30 × 10^9^/L at diagnosis and sufficient hyper-CVAD cycles were prognostic factors for better 4-year OS and LFS, while the B-cell phenotype and higher number of infused CD34^+^ cells in the first cycle were predictors for favorable 4-year LFS. The hyper-CVAD-based MST was a feasible strategy for treating ALL patients with mild toxicity.

## Graphical Abstract

**Graphical Abstract:**
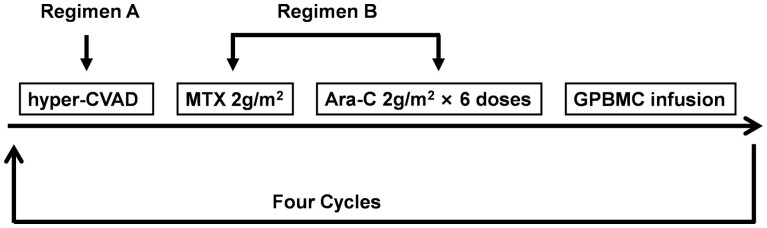

**Lessons Learned**

- Chemotherapy combined with mismatched granulocyte colony-stimulating factor mobilized peripheral blood mononuclear cell infusion (microtransplant) provided a feasible post-remission therapy for patients with acute lymphoblastic leukemia.
- Donor chimerism/microchimerism was detectable in most samples after microtransplant.

**Significance Statement**

The hyper-CVAD-regimen-based microtransplant tended to achieve better leukemia-free survival (LFS) in adults compared with adolescent and young adults (AYA) in acute lymphoblastic leukemia with mild toxicity. Short-term and long-term donor chimerism/microchimerism was detectable in 78% of samples. White blood cell counts at diagnosis, hyper-CVAD cycles received, immunophenotype, and the number of infused CD34^+^ cells in the first cycle had predictive value for overall survival and/or LFS.

## Introduction

Acute lymphoblastic leukemia (ALL) accounts for approximately 30% of acute leukemia with a poor prognosis in adults. Patients usually fail to maintain long-term disease-free survival via multiagent chemotherapy and targeted agents such as tyrosine kinase inhibitors (TKI). Allogeneic stem cell transplant (allo-SCT) raises treatment-related mortality and reduces the quality of life.^1^ Immunotherapeutic strategies such as autologous chimeric antigen receptor-modified T (CAR-T) cell therapy and blinatumomab have shown encouraging outcomes in patients with relapsed and/or refractory B-cell ALL.^2,3^ However, the application of CAR-T cells as post-remission therapy is still controversial concerning over limited antigen exposure. Another consolidation strategy uses CAR-T cells or blinatumomab to eliminate minimal residual disease (MRD) before bridging to allo-SCT.^4,5^ In addition, a chemotherapy-free regimen combining dasatinib with blinatumomab as consolidation has only been explored in Ph chromosome-positive (Ph^+^) ALL patients.^6^

As an alternative immunotherapeutic strategy, we have conducted microtransplant (MST), which combines chemotherapy with mismatched granulocyte colony-stimulating factor (G-CSF) mobilized peripheral blood mononuclear cell (GPBMC) infusion, for patients with acute myeloid leukemia and myelodysplastic syndrome in large cohorts, and both complete remission (CR) and overall survival were improved.^7-9^ For lymphoid malignancies, the hyper-CVAD-based MST protocol for patients with relapsed/refractory lymphoma was reported, and 6 out of 10 patients achieved CR.^10^ In addition, we recently analyzed data from a small cohort of Ph^+^ ALL patients showing favorable outcomes of MST + TKIs over chemotherapy + TKIs and reduced-intensity conditioning (RIC) SCT.^11^ However, the efficacy of MST in the whole ALL population is still unknown. In this study, we analyzed clinical results from patients who received the hyper-CVAD-based MST protocol as post-remission therapy to validate the efficacy of MST in ALL.

## Methods

### Patients and Donors

From July 1, 2009, to January 31, 2018, patients aged 15-68 years who were diagnosed with ALL in remission were screened. Patients who had an available donor were evaluated for eligibility of allogeneic stem cell transplant (allo-SCT). Those with an HLA 10/10 matched related or 9/10-10/10 matched unrelated donor proceeded to allo-SCT. Patients who had a haploidentical donor received MST or haploidentical SCT according to patient choice. Once without any suitable allo-SCT donor, the MST treatment was administered. Collectively, 82 patients were qualified including 14 receiving matched related SCT, 6 receiving 9/10-10/10 matched unrelated SCT, 14 receiving haploidentical SCT, and 48 receiving MST. All those with MST treatment were given a hyper-CVAD-based chemotherapy according to our institution's standard. Among patients receiving MST, 11 with Ph^+^ ALL have been reported in a previous study and were also recruited with extended follow-up. A total of 44 and 4 patients received GPBMC infusion from mismatched related and unrelated donors, respectively. This study was approved by the Human Ethics Committee of The Fifth Medical Center, Chinese PLA General Hospital and was in accordance with the Declaration of Helsinki. All patients and their donors provided written informed consent for receiving MST treatment or collecting and donating GPBMCs before the study.

### Risk Stratification

High-risk ALL was considered in patients with at least one of the following criteria: (1) age >35 years; (2) white blood cell (WBC) count ≥30 × 10^9^/L for B-cell lineage, or ≥100 × 10^9^/L for T-cell lineage; (3) no remission or positive MRD post-induction; and (4) Hypodiploidy (<44 chromosomes), KMT2A (MLL) gene rearrangements, *t*(9;22) chromosomal translocation (BCR-ABL1), complex karyotype (≥5 chromosomal abnormalities), or intrachromosomal amplification of chromosome 21 (iAMP21). The absence of all poor-risk factors is considered a standard risk.

### MST Protocol

Patients received a modified hyper-CVAD regimen for consolidation as previously reported (Supplementary Fig. S1).^11^ In total, 32 out of 48 patients received an additional VMCD regimen at the interval of hyper-CVAD cycles. All patients with Ph chromosome received TKIs. Mismatched GPBMCs were infused 24 h after each completion of middle or high-dose cytarabine. If there were insufficient cells to allow for 4 infusions, additional cycles of the hyper-CVAD regimen without donor cells were still scheduled if the patient was in remission. If relapse occurred during MST treatment, a re-induction therapy was given and the patient had to withdraw from following MST cycles. Patients received subcutaneous G-CSF 5-7 μg/kg/day from the time of neutropenia until neutrophil recovery. No prophylaxis of graft-versus-host disease (GVHD) was given. For Ph^+^ patients, life-long TKIs were given as maintenance. The maintenance regimen of 2-year weekly methotrexate plus daily 6-mercaptopurine was scheduled for Ph^-^ patients.

### Prophylaxis for Central Nervous System Leukemia

The intrathecal injection of methotrexate 15 mg, cytarabine 50 mg, and dexamethasone 5 mg was given during each consolidation cycle. For those who were confirmed to have central nervous system leukemia (CNSL), the intrathecal injection was performed twice a week until clearance.

### Mobilization and Apheresis of Donor PBMCs

Donor PBMC mobilization and apheresis followed our standard protocols as previously reported.^12^ Median numbers of mononuclear, CD34^+^, and CD3^+^ cells infused per cycle in the MST group were 3.05 × 10^8^/kg (range, 1.3-4.03 × 10^8^/kg), 2.20 × 10^6^/kg (range, 0.26-4.17 × 10^6^/kg), and 0.83 × 10^8^/kg (range, 0.36-1.31 × 10^8^/kg), respectively.

### MRD Monitoring

Flow cytometry was used in all patients to determine residual leukemic status at a sensitivity of 0.01%. For patients who preserved fusion genes such as BCR-ABL1, the quantitative RT-PCR assay was used to evaluate MRD status at a sensitivity of 0.001% coupled with the flow cytometry method.

### Donor Chimerism and Microchimerism

The short tandem repeat-polymerase chain reaction (STR-PCR) assay will be performed using peripheral blood if a high proportion of donor chimerism (>1%) was clinically suspected such as sustained hyperpyrexia, rash, diarrhea, and severe hepatic injury following GPBMC infusion. Regardless of the presence of the above symptoms, donor chimerism/microchimerism assay was performed retrospectively for available peripheral blood samples within 14 days after each infusion (short term) and at 3 months or one year after the last infusion (long term) using methods as previously described.^11^

### Criteria for Response and Outcome

CR was defined as less than 5% blasts in the bone marrow without circulating blasts, and neutrophil and platelet counts exceeded 1.0 × 10^9^/L and 100 × 10^9^/L, respectively. Hematologic relapse was defined as leukemic recurrence at any site (>5% blasts if in the bone marrow). Early death was defined as decease within 1 month after study the began. Neutrophil and platelet recovery were defined as the first of 3 consecutive days when counts achieved 0.5 × 10^9^/L and 30 × 10^9^/L, respectively. Definitions of overall survival (OS), leukemia-free survival (LFS), nonrelapse mortality (NRM), acute/chronic GVHD, and severe adverse events were described as previously reported.^11^

### Statistical Analysis

The Kaplan-Meier method and log-rank test were used for survival analyses and survival rate comparison, respectively. Univariate analyses were performed using the log-rank test to identify prognostic variables for OS and LFS. Variables with a *P* value of < .1, as determined by univariate analyses, were considered for entry into multivariate analyses according to Cox proportional hazards regression models. GraphPad Prism (v8.0; GraphPad Software Inc., San Diego, CA) software was used for preparing plots of chimerism/microchimerism. Statistical significance was determined by 2-sided *P* < .05. Statistical packages for the Social Sciences (v25.0; SPSS Inc., Chicago, IL) software was used for statistical analyses.

## Results

### Patient Characteristics

A total of 48 patients were enrolled in this study. The last follow-up was on August 1, 2020. Two (4%) patients were beyond CR1 at enrollment. The median course of the consolidation chemotherapy prior to MST was one (range 0-6). A total of 18 out of 48 patients (38%) finished all 4 MST cycles, and 21 (44%), 7 (15%), and 2 (4%) patients finished 3, 2, and one cycles, respectively. Reasons for fewer cycles are relapse or patient’s refusal of further treatment. Donor cell infusion was given in all cycles except 2 cycles due to insufficient donor cells. A total of 10 out of 11 Ph^+^ patients (91%) received TKI maintenance while 15 out of 37 Ph^−^ patients (41%) entered the maintenance phase of methotrexate plus 6-mercaptopurine. Two patients underwent SCT in remission after MST treatment. MST donors had 0-7 matched HLA loci with paired patients. Characteristics of patients are detailed in Table 1.

**Table 1.** Characteristics of patients.

Parameter	MST (*n* = 48)
Median age (range), years	27 (15-68)
Age <40 year, *n* (%)	35 (73)
Males, *n* (%)	29 (60)
Median WBC (range), × 10^9^/L	10.7 (1.36-327)
WBC ≥30 × 10^9^/L, *n* (%)	11 (23)
Extramedullary leukemia, *n* (%)	19 (40)
Immunophenotype, *n* (%)	
B cell	43 (90)
T cell	5 (10)
Karyotype, *n* (%)	
Normal	34 (71)
Ph-positive	11 (23)
Hypodiploid	1 (2)
Complex karyotype	1 (2)
Other	1 (2)
Risk stratification, *n* (%)	
Standard	19 (40)
High	29 (60)
MRD post induction, *n* (%)	
Negative	33 (69)
Positive	15 (31)
MRD prior to MST, *n* (%)	
Negative	33 (69)
Positive	15 (31)
Median time from diagnosis to MST (range), months	3.2 (1.0-62.6)
Donors, *n*	
5-7 loci matched related	34 (71)
0-4 loci matched related	10 (21)
0-4 loci matched unrelated	4 (8)
Median MNCs per cycle (range), × 10^8^/kg	3.05 (1.30-4.03)
Median CD34^+^ cells per cycle (range), × 10^6^/kg	2.20 (0.26-4.17)
Median CD3^+^ cells per cycle (range), × 10^8^/kg	0.83 (0.36-1.31)

Abbreviations: MNC, mononuclear cells; MRD, minimal residual disease; MST, microtransplant; WBC, white blood cell.

### Hematopoietic Recovery and Toxicity

The median recovery times of neutrophils and platelet were 12 and 14 days, respectively. Severe adverse events included severe infection, nervous system events, and organ failure. Notably, no acute GVHD or early death was present (Table 2).

**Table 2.** Outcomes of patients.

Parameter	MST (*n* = 48)
Median time of ANC recovery (range), days	12 (10-18)
Median time of platelet recovery (range), days	14 (7-20)
Severe adverse event, *n* (%)	
Severe infection	5 (10)
Nervous system events	1 (2)
III-IV aGVHD	0 (0)
Organ failure	2 (4)
Causes of death, *n* (%)	
Relapse	26 (54)
4-year OS, no. of events(%)	18 (62)
Age (year)	
15-39	15 (57)
≥40	3 (76)
Risk stratification	
Standard	8 (58)
High	10 (65)
MRD prior to MST	
Negative	12 (63)
Positive	6 (60)
Immunophenotype	
B cell	16 (63)
T cell	2 (53)
4-year LFS, no. of events (%)	31 (35)
Age (year)	
15-39	26 (26)
≥40	5(62)
Risk stratification	
Standard	14 (26)
High	17 (41)
MRD prior to MST	
Negative	21 (36)
Positive	10 (33)
Immunophenotype	
B cell	27 (37)
T cell	4 (20)
4-year hematologic relapse, no. of events (%)	31 (65)
Age (year)	
15-39	26 (74)
≥ 40	5 (38)
Risk stratification	
Standard	14 (74)
High	17 (59)
MRD prior to MST	
Negative	21 (64)
Positive	10 (67)
Immunophenotype	
B cell	27 (63)
T cell	4 (80)

Abbreviations: ANC, absolute neutrophil count; aGVHD, acute graft-versus-host disease; LFS, leukemia-free survival; MRD, minimal residual disease; MST, microtransplant; OS, overall survival.

### Overall Survival

**Figure 1.**
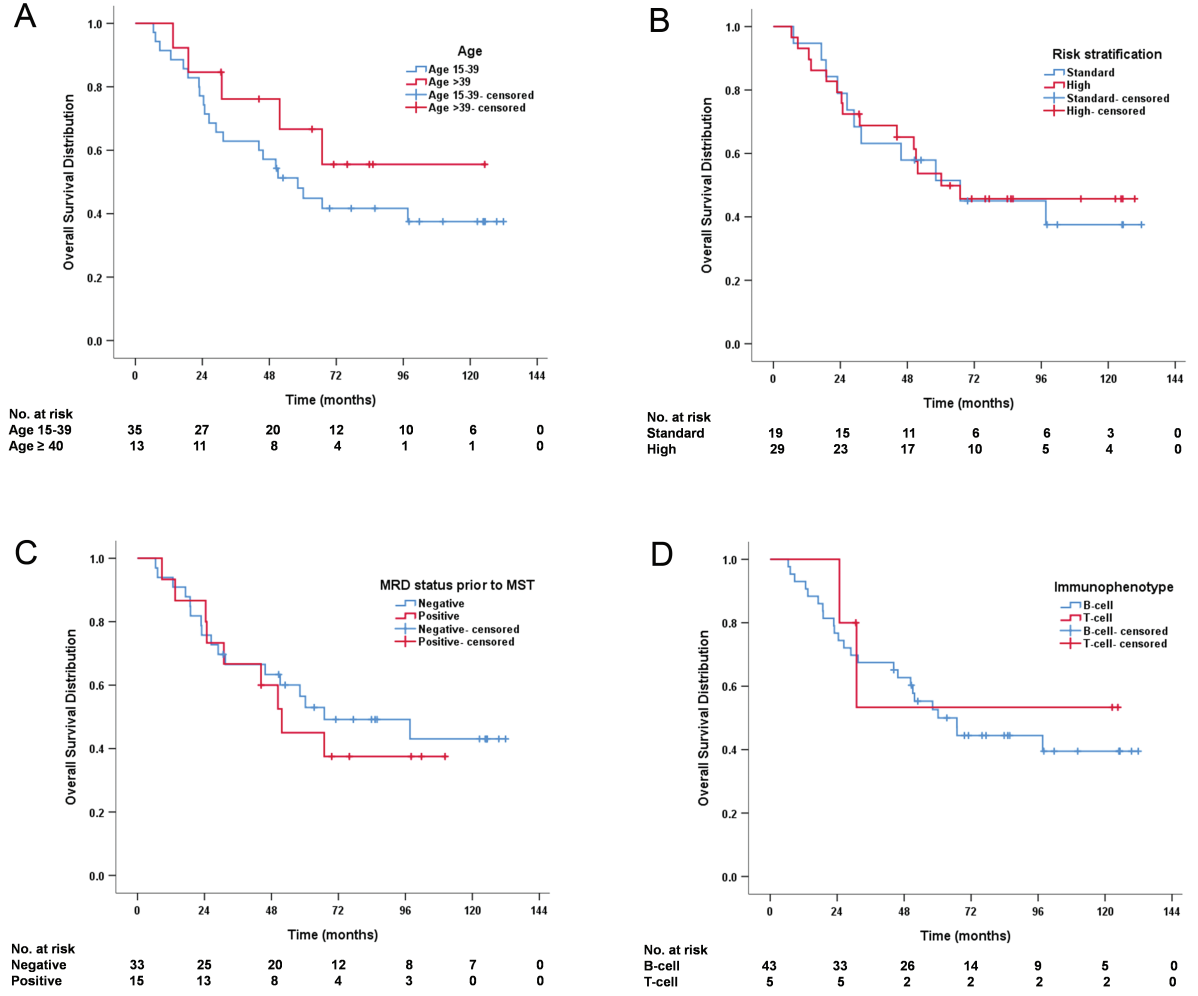
Overall survival (OS) distribution (*n* = 48). (**A**) OS stratified by age. (**B**) OS stratified by risk. (**C**) OS stratified by minimal residual disease prior to MST. (**D**) OS stratified by immunophenotype.

At the last follow-up time, 26 (54%) patients died. All deaths were due to disease progression. The 4-year OS rate was 62% (Table 2). Subgroup analysis was performed stratified by age, risk, MRD status prior to MST, and immunophenotype. Four-year OS was comparable in adolescent and young adult (AYA) patients (15-39 years) and adult patients (≥ 40 years) (57% vs. 76%, *P* = .255) (Fig. 1A). A 4-year OS was also comparable between standard and high-risk cohorts (58% vs. 65%, *P* = .716) (Fig. 1B). When stratified by MRD status prior to MST, similar 4-year OS was observed between MRD-negative and MRD-positive cohorts (63% vs. 60%, *P* = .881) (Fig. 1C). When stratified by the immunophenotype, 4-year OS also had no significant difference between B and T-cell cohorts (63% vs. 53%, *P* = .960) (Fig. 1D).

### Leukemia-Free Survival

**Figure 2.**
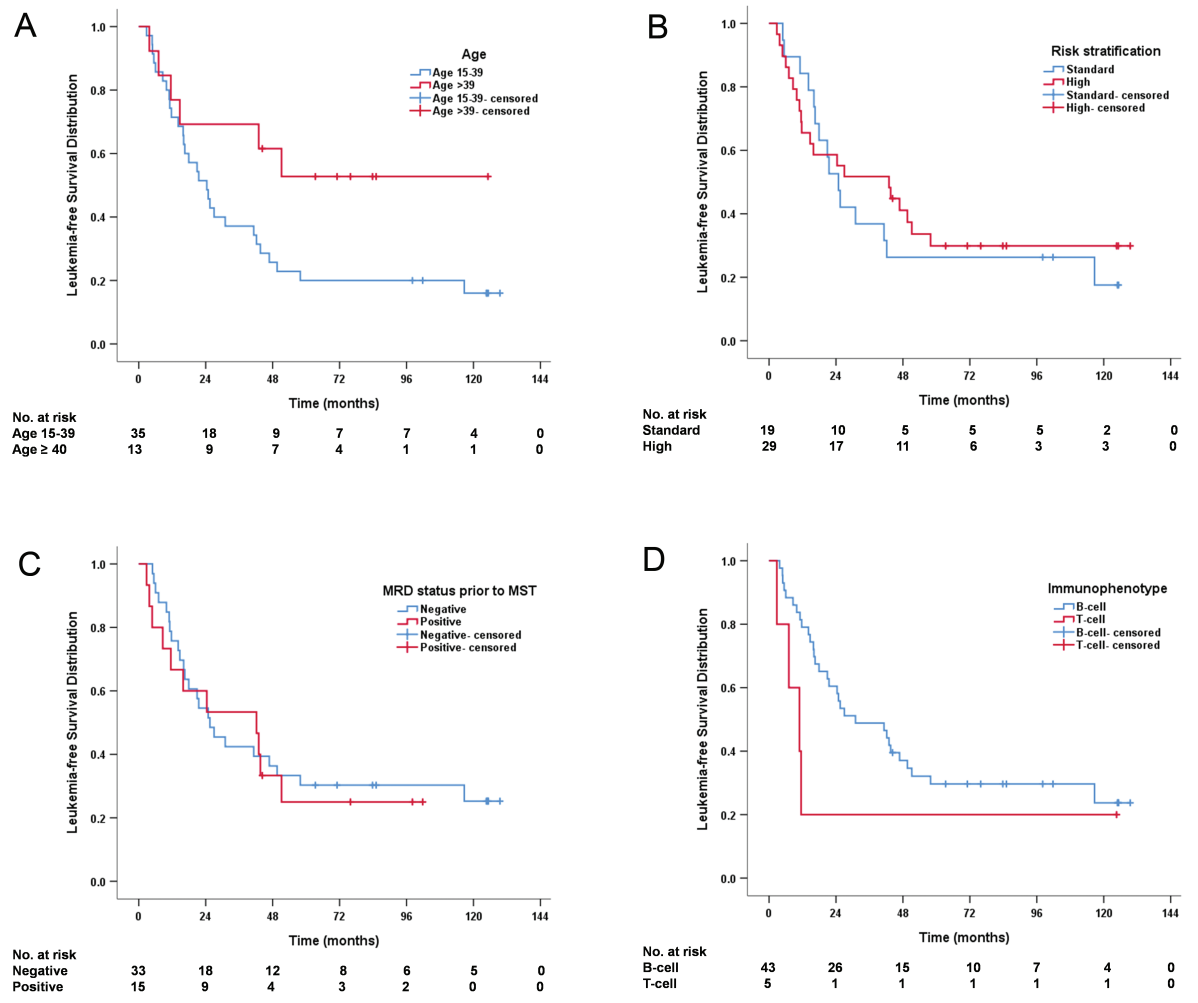
Leukemia-free survival (LFS) distribution (*n* = 48). (**A**) LFS stratified by age. (**B**) LFS stratified by risk. (**C**) LFS stratified by minimal residual disease prior to MST. (**D**) LFS stratified by immunophenotype.

The 4-year LFS rate was 35% (Table 2). Adult patients tended to have better 4-year LFS compared with AYA patients (62% vs. 26%, *P* = .058) despite no statistical significance (Fig. 2A). Comparable LFS was found between standard and high-risk (26% vs. 41%, *P* = .497), MRD-negative and MRD-positive (36% vs. 33%, *P* = .810), and B and T-cell (37% vs. 20%, *P* = .071) cohorts (Fig. 2B-2D).

### Relapse and NRM

**Figure 3.**
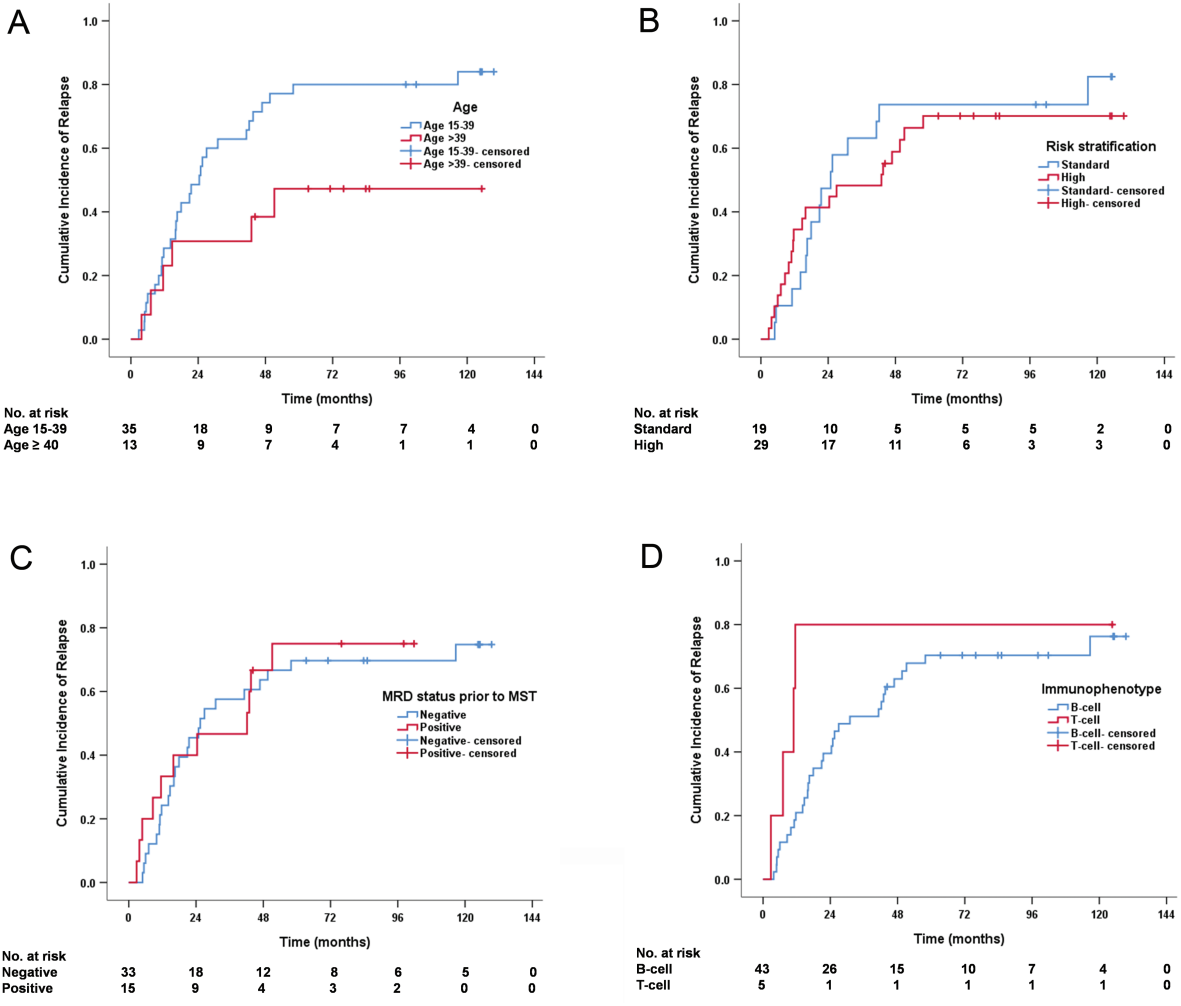
Cumulative incidence of relapse (*n* = 48). (**A**) Relapse stratified by age. (**B**) Relapse stratified by risk. (**C**) Relapse stratified by minimal residual disease prior to MST. (**D**) Relapse stratified by immunophenotype.

The 4-year cumulative incidence of hematologic relapse was 65% (Table 2). Adult patients tended to have lower 4-year relapse rate compared with AYA patients (38% vs. 74%, *P* = .058) despite no statistical significance (Fig. 3A). Similar incidence of hematologic relapse was found between standard and high-risk (74% vs. 59%, *P* = .497), MRD-negative and MRD-positive (64% vs. 67%, *P* = .810), and B and T-cell (63% vs. 80%, *P* = .071) cohorts (Fig. 3B-3D). No patient experienced NRM.

### Donor Chimerism and Microchimerism

**Figure 4.**
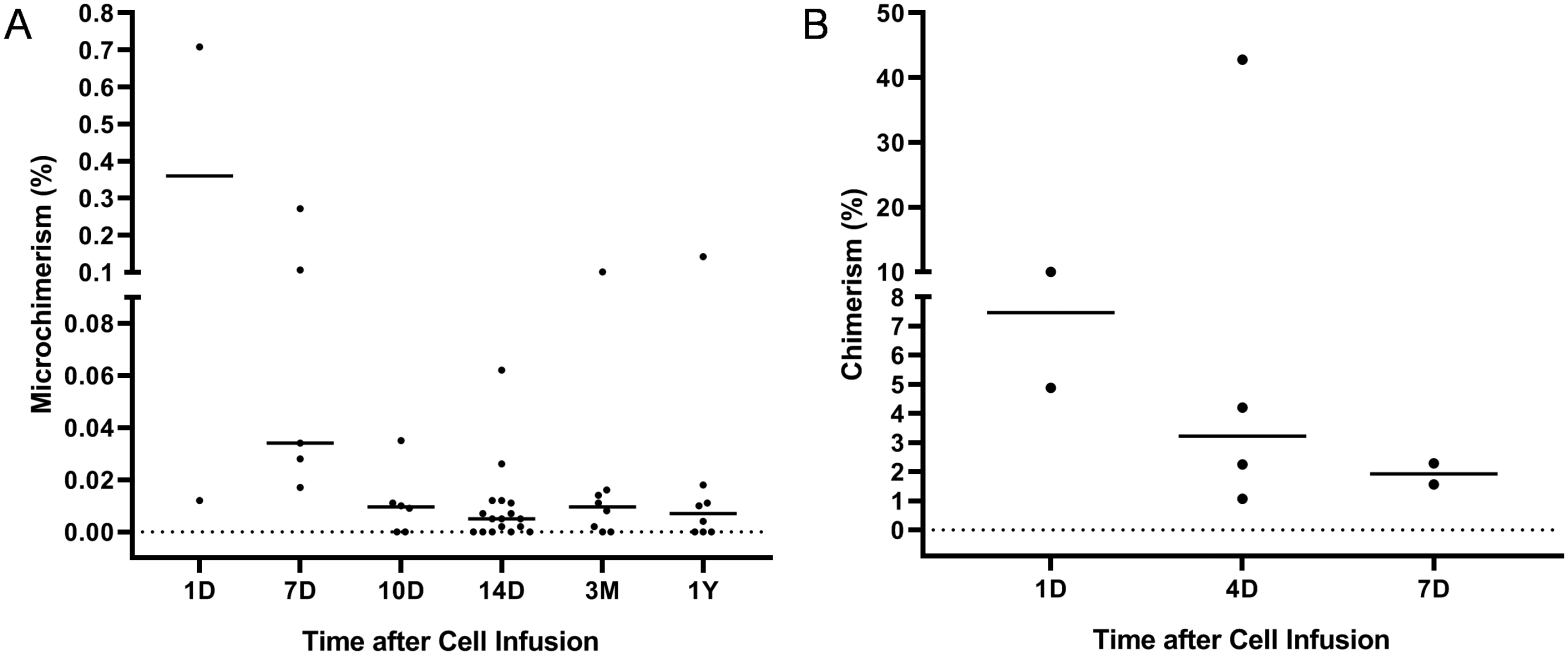
Donor microchimerism and chimerism within 14 days after each infusion (short term) and at 3 months or 1 year after the last infusion (long term) determined by an Indel-primer-based real-time PCR method (*n* = 54). (**A**) Donor microchimerism (*n* = 46). (**B**) Donor chimerism (*n* = 8). D, day; M, month; Y, year.

Sustained hyperpyrexia occurred in 5 infusions and rash was present in 2 infusions. The following STR-PCR assay demonstrated that donor chimerism was detectable with a proportion of 16% on day 6 and turned negative on day 10 after GPBMC infusion in 1 patient with sustained hyperpyrexia and rash. Donor chimerism was undetectable in other infusions with clinical symptoms. In contrast, retrospective analysis using available samples within 14 days after each infusion (short term) and at 3 months or 1 year after the last cell infusion (long term) identified chimerism/microchimerism in 78% (42/54) samples determined by an Indel-primer-based real-time PCR method. For short-term chimerism/microchimerism, the median proportion level decreased over time. Chimerism/microchimerism was detectable in all samples within 7 days ranging from 0.012% to 42.78%, and chimerism >1% was only identified within 7 days ranging from 1.07% to 42.78%. Microchimerism was detectable in 67% (4/6, range: 0.009-0.035%) and 71% (12/17, range: 0.002-0.062%) samples at 10 and 14 days, respectively. For long-term chimerism/microchimerism, no chimerism >1% was identified. The positive rates of microchimerism were 75% (6/8, range: 0.002-0.101%) and 63% (5/8, range: 0.004-0.142%) at 3 months and 1 year, respectively (Fig. 4).

### Univariate and Multivariate Analyses

Patient characteristics (age, gender, WBC at diagnosis, extramedullary leukemia, immunophenotype, risk stratification, MRD prior to MST, and time from diagnosis to MST) and treatment factors (hyper-CVAD cycles received, numbers of HLA-matched loci, MNC, CD34^+^, and CD3^+^ cells infused) were analyzed to identify potential factors influencing OS and LFS (Table 3). On multivariable analysis, white blood cells <30 × 10^9^/L at diagnosis and sufficient hyper-CVAD cycles were prognostic factors for better 4-year OS and LFS, while the B-cell phenotype and number of infused CD34^+^ cells ≥ 2 × 10^6^/kg in the first cycle were predictors for favorable 4-year LFS.

**Table 3.** Univariate and multivariate analyses of factors prognostic for OS and LFS (*n* = 48).

	4-year OS				4-year LFS			
	Univariate analysis		Multivariate analysis		Univariate analysis		Multivariate analysis	
Variables	%	*P*	HR (95% CI)	*P*	%	*P*	HR (95% CI)	*P*
Age								
≤39 years	57.1	.255	–	–	25.7	.058	1.119 (0.369-3.388)	.843
>39 years	76.9		–	–	61.5		1	–
Gender								
Male	65.5	.470	–	–	34.5	.877	–	–
Female	57.9		–	–	36.8		–	–
WBC at diagnosis								
<30 × 10^9^/L	73.0	.004	0.351 (0.136-0.909)	.031	43.2	.012	0.362 (0.158-0.831)	.016
≥30 × 10^9^/L	27.3		1	–	9.1		1	–
Extramedullary leukemia								
Yes	52.6	.300	–	–	31.6	.480	–	–
No	69.0		–	–	37.9		–	–
Immunophenotype								
B cell	62.8	.960	–	–	37.2	.071	0.292 (0.087-0.979)	.046
T cell	60.0		–	–	20.0		1	–
Risk stratification								
Standard	57.9	.716	–	–	26.3	.497	–	–
High	65.5		–	–	41.4		–	–
MRD prior to MST								
Negative	63.6	.881	–	–	36.4	.810	–	–
Positive	60.0		–	–	33.3		–	–
Time from diagnosis to MST								
≥3 months	59.3	.687	–	–	25.9	.142	–	–
<3 months	66.7		–	–	47.6		–	–
Hyper-CVAD cycles received								
4	94.4	.001	0.090 (0.012-0.691)	.021	66.7	<.001	0.183 (0.068-0.494)	.001
<4	43.3		1	–	16.7		1	–
HLA-matched loci								
0-4	42.9	.059	1.996 (0.776-5.135)	.152	21.4	.077	2.086 (0.937-4.648)	.072
5-7	70.6		1	–	41.2		1	–
Mononuclear cells infused in the first cycle								
≥3 × 10^8^/kg	57.1	.499	–	–	35.7	.905	–	–
<3 × 10^8^/kg	70.0		–	–	35.0		–	–
CD34^+^ cells infused in the first cycle								
≥2 × 10^6^/kg	66.7	.429	–	–	48.1	.067	0.367 (0.164-0.823)	.015
<2 × 10^6^/kg	57.1		–	–	19.0		1	–
CD3^+^ cells infused in the first cycle								
≥0.8 × 10^8^/kg	64.3	.544	–	–	35.7	.708	–	–
<0.8 × 10^8^/kg	60.0		–	–	35.0		–	–

Abbreviations: LFS, leukemia-free survival; MRD, minimal residual disease; MST, microtransplant; OS, overall survival; WBC, white blood cell.

## Discussion

Post-remission therapy for patients with ALL is still controversial due to the heterogeneity of ALL. The choice depends on various factors such as age, risk stratification, MRD status, and cytogenetic features. Although matched allogeneic SCT is recommended for fit patients with high-risk features or positive MRD status, the high NRM seems to neutralize its low relapse rate, leading to a similar OS compared with chemotherapy. Therefore, multiagent chemotherapy is still a consideration.^13,14^ In this study, we aimed to explore the safety and efficacy of the hyper-CVAD regimen combined with GPBMC infusion in a large cohort. No acute GVHD, early death, or NRM occurred. OS rates were comparable between groups with different ages, risk stratification, MRD status prior to MST, and immunophenotype. Adult patients tended to have better 4-year LFS and lower hematologic relapse rates compared with AYA patients. Short-term and long-term donor chimerism/microchimerism was detectable in 78% of samples. WBC counts at diagnosis, hyper-CVAD cycles received, immunophenotype, and the number of infused CD34^+^ cells in the first cycle had predictive values for OS and/or LFS.

Patients using the hyper-CVAD regimen as induction and consolidation had both 4-year survival and CR duration rates of approximately 40%.^15^ For the young CD20-positive cohort, 4-year survival, and CR duration rates increased to approximately 65% and 60% when adding rituximab into the hyper-CVAD regimen.^16^ In this study, the 4-year OS was 62%, comparable to the rituximab-containing regimen and better than the hyper-CVAD regimen alone. However, no benefit of 4-year LFS was observed compared with either cohort above, mainly resulting from a high relapse rate. This may be partly attributed to the low proportion of patients entering the maintenance phase in Ph^−^ patients because significant OS and LFS differences were observed between cohorts with and without maintenance (4-year OS 87% vs. 23%, *P* < .001 and 4-year LFS 47% vs. 0%, *P* < .001) (Supplementary Fig. S2). Nonetheless, these differences could also be explained that relapse during the consolidation phase reduced chance to go into maintenance. Outcomes in the AYA subgroup also showed that the 4-year OS was 57%, superior to that using the adult CALGB regimen but inferior to that using the pediatric CCG protocol. Similar to the entire cohort, no favorable 4-year LFS was observed in the AYA group.^17^ In contrast, the 4-year OS achieved 76% in adult patients over 40 years old who received hyper-CVAD-based MST (Table 2). These results demonstrated that the hyper-CVAD-based MST protocol appeared to gain more survival benefits in adult rather than AYA patients, implying that the hyper-CVAD regimen may not be the most desirable protocol combined with MST for AYA patients. Pediatric-inspired MST regimens may improve outcomes in AYA patients deserving further study.^18^ It should also be noted that among 13 adult patients, 7 (54%) were Ph^+^. In contrast, only 4 out of 35 (11%) AYA patients were Ph^+^. This is consistent with previous reports that the frequency of BCR-ABL1 rearrangement increases with age.^19^ Moreover, Ph^+^ patients substantially benefited from TKIs especially TKIs plus MST according to our previous report.^11^ That may also explain the survival distinction further highlighting the effect of targeted therapy in the non-transplant situation. In addition, adult patients may benefit more from the absence of early death and NRM via MST because elderly patients generally undergo more severe chemotherapy-related toxicities compared with young patients.

The prognosis of patients in different risk groups is usually varied. In the MRC UKALL XII/ECOG E2993 study, patients were stratified into standard and high-risk groups according to clinical features. The 5-year OS rates were 57% and 35%, respectively, in standard and high-risk groups who achieved CR.^20^ When incorporated with cytogenetics, the impact of risk stratification on prognosis was even significant.^21^ In this study, we stratified patients according to both clinical features and cytogenetics. Notably, no survival difference was found between standard and high-risk cohorts in patients receiving MST. This may be partly due to the favorable survival outcome of MST for the Ph^+^ cohort as previously described, which was defined as high risk in the pre-TKI era.^11^ Stratification by Ph chromosome status in the MST group in this study also showed improved 4-year OS in the Ph-positive cohort (91% *vs.* 54%, P =.048) (Supplementary Fig. S3).

The MRD-directed treatment option was widely accepted. For patients who achieved MRD negativity, it is recommended to receive either consolidation therapy or allo-SCT. However, for those with MRD, it is still controversial whether to initiate allo-SCT with MRD or give bridging therapies to achieve MRD negativity.^6,14^ This study also showed that either OS or LFS was comparable regardless of MRD status prior to MST, indicating that MST may give as either consolidation therapy for MRD-negative patients or bridging therapy for MRD-positive patients. Nonetheless, allo-SCT should also be evaluated for MRD-positive patients when achieving MRD negativity through bridging treatment due to the relatively high relapse rate of MST.

Results of chimerism/microchimerism after MST vary due to distinct detection methods and time points in previous studies. A study including 22-time points after MST detected no donor microchimerism using a real-time HLA-polymorphism specific quantitative PCR assay.^22^ However, another study using a highly sensitive droplet digital PCR assay detected microchimerism in most samples following MST or third-party cellular therapies.^23^ In this study, donor chimerism/microchimerism was also detectable in most samples in either short-term or long-term following GPBMC infusion, which indicates the persistence of donor components. In addition, another study using cord blood-derived adoptive cell therapy for refractory AML demonstrated that chimerism was a powerful predictor of clinical response.^24^ However, we were unable to determine the correlation between chimerism/microchimerism and response in this study due to a lack of scheduled sample collection which warrants further research in the future.

## Conclusion

The hyper-CVAD-based MST is feasible in treating adult ALL patients with mild toxicity. Relapse was still a concern to decrease LFS. Limitations of this study lie in the retrospective property, which lacks correlative studies including scheduled tests of microchimerism and immune function to explore mechanisms. The fact that fewer adults than AYA, fewer patients with T-cell than B-cell phenotype, and low proportion of finishing all 4 MST cycles may also lead to an unbalanced comparison and non-significant outcomes. Prospective studies to compare MST with multiagent chemotherapy in standard-risk patients or with SCT in high-risk patients may better demonstrate the role of GPBMC infusion. Further studies may also extend to MST combining with other chemotherapy regimens such as pediatric-inspired protocols as well as immunotherapeutics and targeted agents such as tisagenlecleucel, blinatumomab, and next-generation TKIs.

## Supplementary Material

szac066_suppl_Supplementary_Material

## Data Availability

The data that support the findings of this study are available from the corresponding author upon reasonable request.
