# Supplementary material for: Hyper-CVAD-Based Stem Cell Microtransplant as Post-Remission Therapy in Acute Lymphoblastic Leukemia
Source: Stem Cells Transl Med. 2022 Oct 1;11(11):1113–22. doi: 10.1093/stcltm/szac066 (PMC9672851; doi:10.1093/stcltm/szac066)
Supplement: szac066_suppl_Supplementary_Material [file szac066_suppl_supplementary_material.docx]

**Supporting files**

**Hyper-CVAD-based stem cell microtransplant as post-remission therapy in acute lymphoblastic leukemia**

Bo Cai^1#^; Yi Wang^1#^; Yangyang Lei^1#^; Yanping Shi^1#^; Qiyun Sun^1^; Jianhui Qiao^1^; Kaixun Hu^1^; Yaqing Lei^1^; Bingxia Li^1^; Tieqiang Liu^1^; Zhiqing Liu^1^; Bo Yao^1^; Xuecong Zhao^1^; Xiaofei Li^1^; Wen Zhao^1^; Xiujie Feng^1^; Anli Xie^1^; Xin Ning^1^; Mingxing Feng^1^; Weiwei Zhao^1^; Jiayue Guo^2^; Huisheng Ai^1^; Changlin Yu^1*^; Mei Guo^1*^

^1^Department of Hematology, The Fifth Medical Center, Chinese PLA General Hospital, Beijing, China

^2^Department of Clinical Medicine, Capital Medical University, Beijing, China

^#^These authors contributed equally to this work.

^*^Corresponding authors

**supporting Figures**


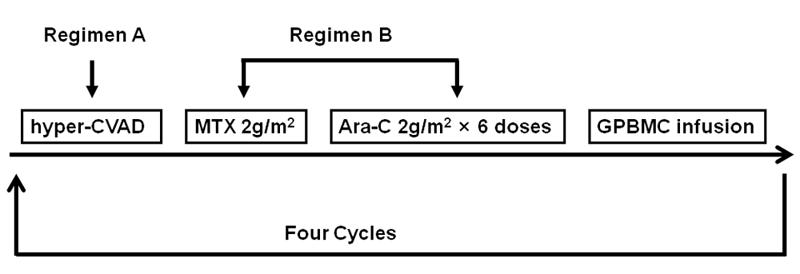


**Figure S1.** The protocol of microtransplant. The hyper-CVAD regimen A consists of fractionated cyclophosphamide 300 mg/m^2^ every 12 hours on days 1-3, vincristine 2 mg on day 1, doxorubicin 50 mg/m^2^ on day 4 and dexamethasone 40 mg/d on days 1-4. The dose of cytarabine (Ara-C) reduced to 1 g/m^2^ for patients aged ≥ 60 years. G-CSF mobilized peripheral blood mononuclear cells (GPBMC) were infused 24 hours after each completion of cytarabine. MTX, methotrexate.


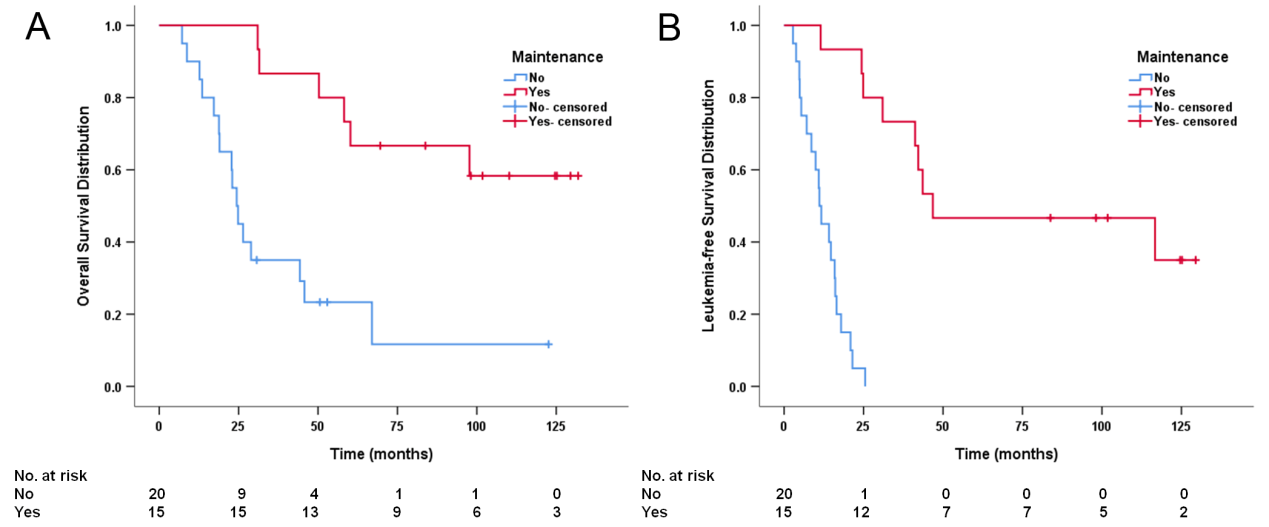


**Figure S2.** Overall survival (OS) and leukemia-free survival (LFS) in Ph^-^ patients with and without methotrexate plus 6-mercaptopurine maintenance (n = 35). Two patients who received transplant in remission were excluded from analysis. (A) OS distribution. (B) LFS distribution.


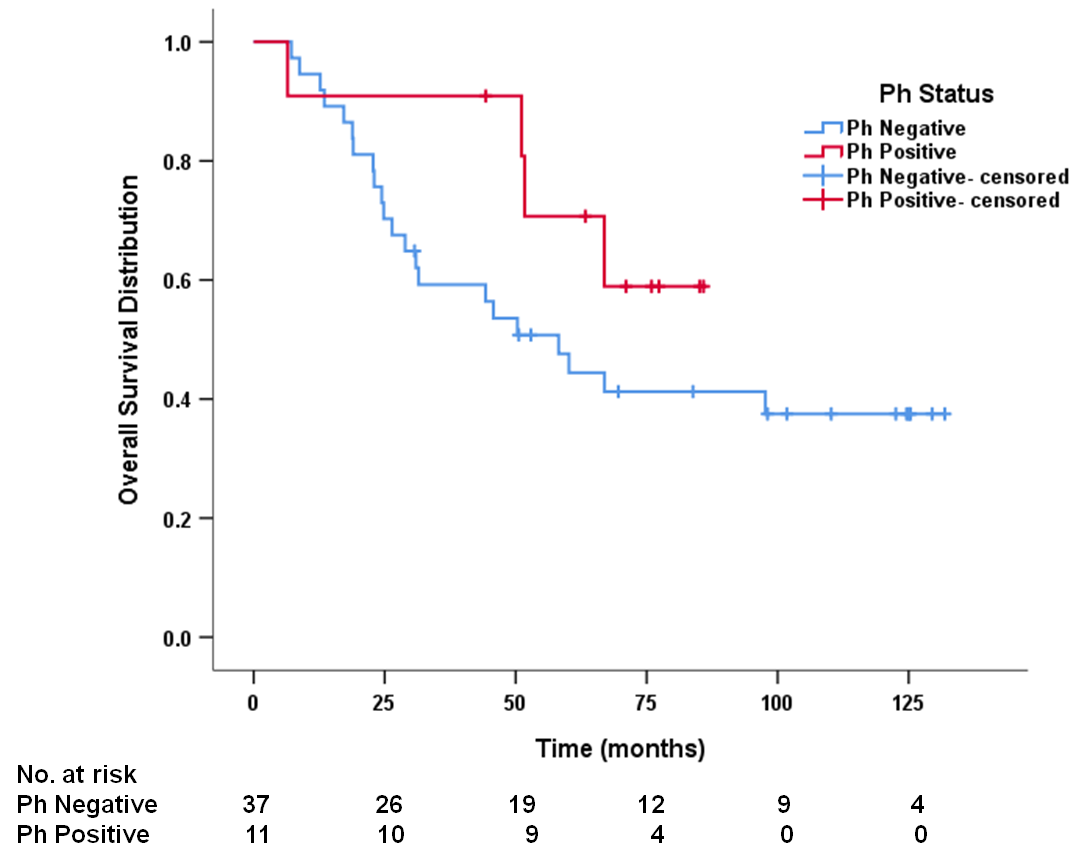


**Figure S3.** Overall survival stratified by Ph chromosome status (n = 48).
